# Dysfunction of Mitochondrial Dynamics in *Drosophila* Model of Diabetic Nephropathy

**DOI:** 10.3390/life11010067

**Published:** 2021-01-18

**Authors:** Kiyoung Kim, Sun Joo Cha, Hyun-Jun Choi, Jeong Suk Kang, Eun Young Lee

**Affiliations:** 1 Department of Medical Biotechnology, Soonchunhyang University, Asan 31538, Korea; 2 Department of Medical Sciences, Soonchunhyang University, Asan 31538, Korea; cktjswn92@sch.ac.kr; 3 Department of Integrated Biomedical Sciences, Soonchunhyang University, Cheonan 31151, Korea; chj5913@sch.ac.kr; 4 Division of Nephrology, Department of Internal Medicine, Soonchunhyang University Cheonan Hospital, Cheonan 31151, Korea; abalonea@naver.com; 5 Institute of Tissue Regeneration, College of Medicine, Soonchunhyang University, Cheonan 31151, Korea; 6 BK21 FOUR Project, College of Medicine, Soonchunhyang University, Cheonan 31151, Korea

**Keywords:** diabetic nephropathy, *Drosophila*, mitochondrial dynamics, nephrocyte, disease model

## Abstract

Although mitochondrial dysfunction is associated with the development and progression of diabetic nephropathy (DN), its mechanisms are poorly understood, and it remains debatable whether mitochondrial morphological change is a cause of DN. In this study, a *Drosophila* DN model was established by treating a chronic high-sucrose diet that exhibits similar phenotypes in animals. Results showed that flies fed a chronic high-sucrose diet exhibited a reduction in lifespan, as well as increased lipid droplets in fat body tissue. Furthermore, the chronic high-sucrose diet effectively induced the morphological abnormalities of nephrocytes in *Drosophila*. High-sucrose diet induced mitochondria fusion in nephrocytes by increasing Opa1 and Marf expression. These findings establish *Drosophila* as a useful model for studying novel regulators and molecular mechanisms for imbalanced mitochondrial dynamics in the pathogenesis of DN. Furthermore, understanding the pathology of mitochondrial dysfunction regarding morphological changes in DN would facilitate the development of novel therapeutics.

## 1. Introduction

Diabetic nephropathy (DN) is the most common cause of progressive kidney disease associated with diabetes mellitus, characterized by the presence of urine albumin excretion, diabetic glomerular lesions, and podocyte loss [1,2]. The pathogenic mechanisms of DN have not been clearly elucidated. To understand the exact molecular mechanisms of DN pathogenesis, various studies have used high-sucrose or high-lipid diet models [3,4,5,6]. From these studies, it is clear that high-sucrose or high-lipid diets induce various defective phenotypes: hyperglycemia, lipid accumulation, mitochondrial dysfunction, and increased reactive oxygen species (ROS) [5,7,8]. Recent studies have suggested that podocytes are the pathogenic target cells to prevent albuminuria and preserve renal function in DN. Podocytes are epithelial cells, localized on the urinary side of the glomerular barrier, and they encircle the glomerular capillaries [9,10]. They wrap around the glomerular capillaries to form the foot processes that provide the selective slit diagram for glomerular filtration. Therefore, there is an urgent need to investigate the relationship between chronic diet-induced metabolic disorders and podocyte dysfunction in the development and progression of DN.

Mitochondria play an essential role in the survival of all cells responsible for cellular respiration, ROS generation, and adenosine triphosphate(ATP) production by oxidative phosphorylation [11]. Previously, it has been reported that mitochondrial membrane potential in endothelial cells and podocytes of the kidney decreased in DN [12,13]. Highly increased ROS generation by mitochondria plays a role in metabolic pathways, including inflammation and apoptosis, in response to hyperglycemia [14,15]. Furthermore, several reports have described enlarged mitochondria in podocytes of a DN rat model [16,17]. From these studies, emerging evidence indicates that the disruption of mitochondrial function and morphology may be critical for the development and progression of DN.

This study investigated the effects of a chronic high-sucrose diet using the *Drosophila* model. *Drosophila* nephrocytes have been suggested to be structurally and functionally analogous to vertebrate podocytes in kidney [18,19]. *Drosophila* nephrocyte abnormality in flies, as in vertebrates, was found to be associated with a chronic high-sucrose diet. Moreover, it was found that a chronic high-sucrose diet induced mitochondrial fusion in nephrocytes by increasing Opa1 and Marf expression. This finding provides novel evidence for the molecular link between Opa1/Marf expression and diabetic nephropathy pathogenesis in vivo. These findings raise the prospect of targeting the regulators of imbalanced mitochondrial dynamics as a putative therapeutic strategy in the pathogenesis of DN.

This study further highlights the utility of the *Drosophila* in vivo model for imbalanced mitochondrial dynamics associated with a chronic high-sucrose diet for investigating DN pathomechanisms and for drug discovery through natural or chemical compound screening in a clinically relevant and experimentally tractable model. However, further studies are needed to utilize *Drosophila* genetics to identify novel regulation factors and signaling pathways, with respect to the mitochondrial dynamics involved in the development and progression of DN.

## 2. Materials and Methods

### 2.1. Drosophila Stocks

All stock flies were raised at 25 °C on standard food. Crosses were performed using a standard procedure. The *UAS-mCD8-RFP* and *UAS-mitoGFP* lines were obtained from the Bloomington *Drosophila* Stock Center. The podocyte-specific driver *snsGCN-Gal4* line was provided by Dr. Ross L. Cagan (University of Pennsylvania, Philadelphia, PA, USA).

### 2.2. Lifespan Assay

Twenty male flies (total *n* > 100) were collected and transferred into different vials containing fly food with or without 1 M sucrose mixed at 25 °C. All groups of flies were transferred to fresh vials every other day. The number of dead flies was recorded daily.

### 2.3. Immunohistochemistry

Adult fly abdomens were dissected from flies and fixed with 4% formaldehyde in a fixative buffer (100 mM 1,4-piperazinediethanesulfonic acid (PIPES), 1 mM ethylene glycol tetraacetic acid (EGTA), 1% Triton X-100, and 2 mM MgSO_4_, pH 6.9) for 30 min at 25 °C. For Nile Red staining, flies were washed in phosphate-buffered saline with Tween 20 (PBST) and incubated for 30 min in Nile Red (Sigma-Aldrich, Cat#: M3013, St. Louis, MO, USA) staining solution (1 mg/mL in dimethyl sulfoxide, 1:2000 in PBST). The samples were washed in PBST and mounted in *SlowFade^TM^* Gold antifade reagent (Invitrogen, Cat#: S36936, Carlsbad, CA, USA).

For analysis of nephrocyte morphology, dissected abdomens were fixed with a fixative buffer for 30 min and washed in PBST. The samples were mounted in an antifade reagent. All images were collected using a Leica fluorescence microscope (MZ10F) (Leica Microsystems, Wetzlar, Hessen, Germany) and a Carl Zeiss confocal microscope (LSM710) (Carl Zeiss, Oberkochen, Baden-Württemberg, Germany).

### 2.4. Western Blot Analysis

Protein extracts were prepared by homogenizing ten 25-day-old male fly abdomens. The total protein extracts (10 μg) were separated using a 4–12% gradient SDS-PAGE gel and transferred to polyvinylidene difluoride membranes (Millipore, Cat#: 10600023, Burlington, MA, USA). The membranes were blocked with Tris-buffered saline with 4% nonfat dry milk or 4% bovine serum albumin for 1 h. The following primary antibodies were used: rabbit anti-Opa1 (1:500; Sigma-Aldrich, Cat#: M6319), rabbit anti-Marf (1:2000; a gift from Alexander Whithworth, University of Cambridge), and rabbit anti-β-actin (1:4000; Cell Signaling Technology, Cat#: 4967, Danvers, MA, USA). The primary antibodies were detected with the following horseradish peroxidase (HRP)-conjugated secondary antibodies: goat anti-rabbit IgG HRP conjugate (1:2000; Millipore, Cat#: AP307P) and goat anti-mouse IgG HRP conjugate (1:2000; Millipore, Cat#: AP308P). Detection was carried out using an ECL-Plus kit (Amersham, Cat#: RPN2232, Amersham, Buckinghamshire, United Kingdom).

## 3. Results and discussion

### 3.1. Flies Fed Chronic High-Sucrose Diet Displayed Phenotypes of Previous Drosophila Models of Diabetes

Flies fed a high-sucrose diet exhibited increased lipid and carbohydrates, indicative of an obese- and diabetes-like phenotype [5]. A high-sucrose diet induced severe hyperglycemia and insulin resistance in *Drosophila* [5,6,20,21,22]. Therefore, *Drosophila* has been proven to be a good model for studying type 2 diabetes as well as diabetes complications. DN is a chronic and progressive kidney disease associated with diabetes mellitus, characterized by defects in the glomerular basement membrane, glomerular endothelial cell injury, and podocyte loss [1]. Despite several studies using animal models of DN, the mechanisms underlying sucrose diet-induced nephrocyte dysfunction remain unclear. Thus, there is a need to find novel regulators and therapeutics to prevent DN development and progression using in vivo studies.

**Figure 1:**
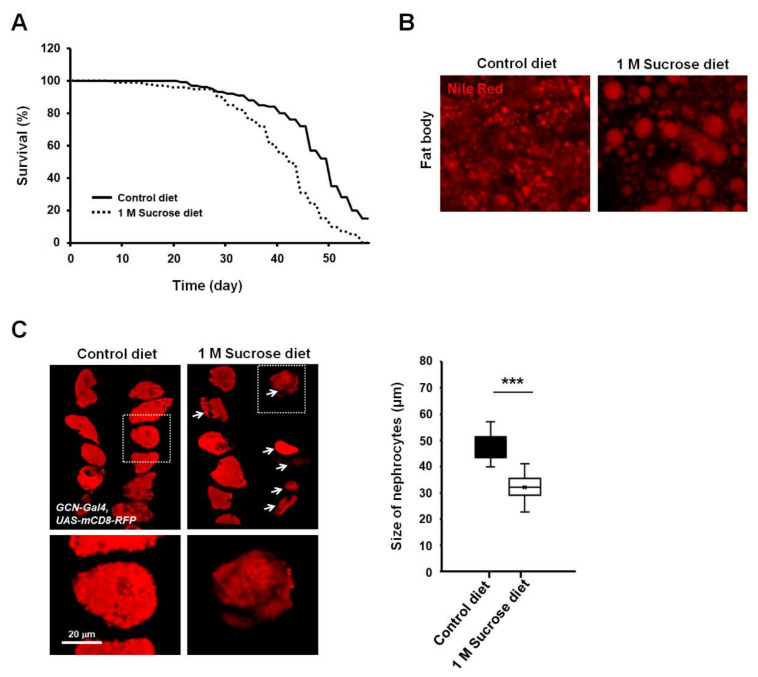
High-sucrose diet reduces lifespan and increases lipid droplet size in *Drosophila*. (**A**) Effect of high-sucrose diet on the lifespan of adult flies. High-sucrose diet of 1 M sucrose was fed to each fly for 8 weeks (*n* ≥ 100). (**B**) Nile Red staining of the dissected adult abdomens from 28-day-old adult flies. Enlarged lipid droplets are visible in the fat body of sucrose-treated adult flies. (**C**) A high-sucrose diet reduces the size of nephrocytes in *Drosophila*. Expression of mCD8-RFP in nephrocytes significantly decreased in flies fed a chronic high-sucrose diet. Quantification of nephrocyte size difference between flies fed control diet versus high-sucrose diet. Each measurement represents the average size of 20–30 cells. Error bars represent mean ± standard deviation of three independent experiments. The experimental significance was determined using the Student’s *t*-test (***, *p* < 0.001).

To determine the effects of chronic high-dose sucrose diet on lifespan in *Drosophila*, flies were fed with a regular medium mixed with or without 1 M sucrose, and longevity was monitored. Consistent with previous reports, our findings also indicated a significant reduction in lifespan in high-sucrose diet flies (Figure 1A). High-sucrose or high-fat diet induced dysregulation of lipid and carbohydrate metabolism. Recently, many researchers have observed increased triacylglycerol levels in mice and *Drosophila* fed with high doses of sucrose [5,6]. The fat body of *Drosophila* is a functional homolog of both the liver and adipose in animals and is a well-known tissue of stored fat [23]. To further investigate whether a high-sucrose diet increased fat storage in fat body tissue, lipid droplets of the adult fat body were stained with Nile Red. Results showed that the lipid droplets of fat body in flies fed a chronic high-sucrose diet were significantly larger than those in control-fed animals (Figure 1B).

To assess whether the chronic high-sucrose diet affects nephrocyte functions in *Drosophila*, we labeled nephrocytes using a membrane-bound red fluorescent protein (mCD8-RFP) reporter driven by a nephrocyte-specific driver, sns-GCN-Gal4, and chronically fed flies with 1 M sucrose for 4 weeks. Interestingly, the average size of nephrocytes strongly decreased in high-sucrose-treated flies compared to those in control flies (Figure 1C, arrows). Furthermore, the density of mCD8 signals in nephrocytes was decreased in flies fed a chronic high-sucrose diet (Figure 1C, magnification views). These results indicated that a chronic high-sucrose diet caused severe morphological abnormalities in the nephrocytes of *Drosophila*.

### 3.2. Chronic High-Sucrose Diet Induced Mitochondrial Dynamic Imbalance

Mitochondria are critical intracellular organelles involved in various biological activities, including ATP production, ROS generation, apoptosis, and calcium homeostasis [24]. Furthermore, mitochondria are highly dynamic organelles, regulated by mitochondrial fusion and fission processes. The balance between these two processes regulates mitochondrial morphology, size, number, and location within the cytoplasm [25]. Mitochondrial fusion is the union of two mitochondria resulting in one mitochondrion, whereas mitochondrial fission is characterized by the division of one mitochondrion into two daughter mitochondria [26]. Most cells maintain a specific mitochondrial morphology, ranging from fragmented to fused shape under different metabolic conditions [27]. Additionally, imbalance of mitochondrial dynamics can result in several common diseases, including cancer and neurodegenerative diseases, and may strongly affect disease pathogenesis [28]. The kidney is by far the second highest consumer of oxygen in our body [29]. Oxygen is metabolized in the mitochondria via oxidative phosphorylation to produce ATP. Therefore, mitochondria may play crucial roles in maintaining kidney function. Recent studies have indicated that mitochondrial dysfunction is involved in the development and progression of DN [30,31]. DN is associated with reduced mitochondrial membrane potential and excessive mitochondrial ROS production in nephrocytes. Human patients with DN have impaired mitochondrial metabolism in diabetic kidneys [2,13]. Furthermore, it is important to know that some of the different aspects of mitochondrial physiology can regulate mitochondrial morphological changes and dynamics. In the 1990s, enlarged mitochondria were first reported in proximal tubule cells of patients with diabetes and correlated with microalbuminuria in a rat model of diabetes [16,17]. This evidence suggests that imbalanced mitochondrial dynamics may be critical in the pathogenesis of DN. However, the molecular mechanisms that regulate mitochondrial dynamics in the pathogenesis of DN are not fully elucidated yet. Moreover, further studies that identify the novel regulators that cause an imbalance of mitochondrial dynamics in the development of DN, as well as studies that establish new in vivo models, are needed.

**Figure 2:**
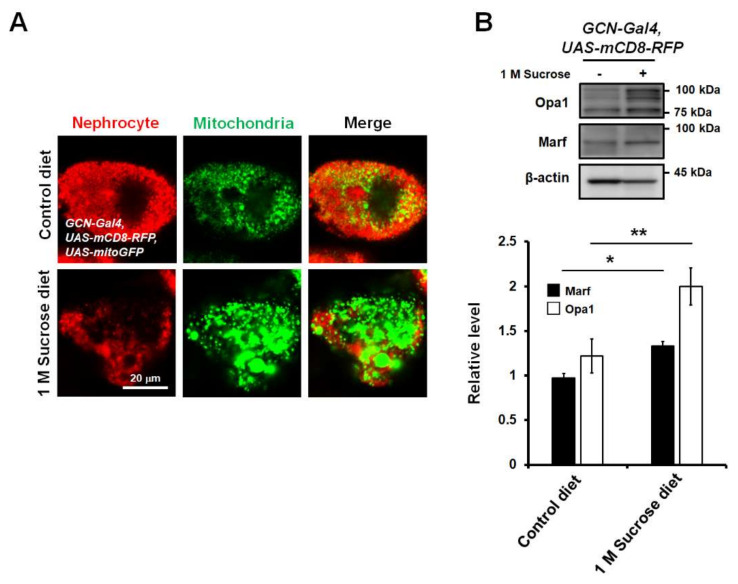
High-sucrose diet increases mitochondrial fusion in nephrocytes of *Drosophila*. (**A**) High-sucrose diet-induced abnormal shape and size of nephrocytes in *Drosophila* (mCD8-RFP). Mitochondria were visualized using mitochondria-targeted green fluorescent protein (mito-GFP). Mitochondrial morphology was highly fused in nephrocytes of chronic high-sucrose diet flies. (**B**) Opa1 and Marf protein levels in abdomen extracts from high-sucrose diet flies. Opa1 and Marf levels in abdominal tissues were significantly increased in chronic sucrose-treated adult flies. β-actin was used as a loading control. Both Opa1 and Marf expression levels were normalized to that of β-actin. Statistical significance was determined using the Student’s *t*-test (*, *p* < 0.05; **, *p* < 0.01). Error bars represent mean ± standard deviation of three independent experiments.

To develop a new in vivo model for the study of diabetes-induced mitochondrial dysfunction in nephrocytes, to study whether a high-sucrose diet regulates mitochondrial dynamics in the nephrocytes of *Drosophila*, and to observe mitochondrial morphological changes, we crossed flies overexpressing both mCD8-RFP and mitochondria-targeted green fluorescent protein (mito-GFP) with *sns-GCN-Gal4*. Due to the specific expression of mito-GFP in nephrocytes, mitochondria were easily visualized using a fluorescent confocal microscope. Notably, enlarged mitochondria were observed in the nephrocytes of flies fed a chronic high-sucrose diet (Figure 2A), indicating that mitochondria in high-sucrose diet flies exhibited increased mitochondrial fusion. The enlarged mitochondrial morphology in the high-sucrose diet may be due to the activation of fusion proteins. The machinery for mitochondrial fusion and fission is composed of dynamin-like GTPases including mitofusin 1/2 (MFN1/2; Marf, *Drosophila* homologue), optic atrophy 1 (Opa1), mitochondrial fission 1 protein (Fis1), and dynamin-related protein 1 (Drp1). Mitochondrial fusion is promoted by MFN1/2, which is required for outer membrane fusion, and Opa1, which is essential for inner membrane fusion [32]. Drp1 mediates fission of the outer mitochondrial membrane [26]. Thus, to understand how high-sucrose diet affects mitochondrial morphology, we checked the expression levels of key regulatory elements of the mitochondrial fusion–fission machinery that controls mitochondrial dynamics in the abdominal tissues of flies fed a high-sucrose diet. Total protein was extracted from the abdominal tissues of flies and Western blotting was performed to analyze the protein levels of various regulatory proteins in flies fed a high-sucrose diet compared to those in control diet flies. Because there are no good antibodies for *Drosophila* Drp1 and Fis1, only Opa1 and Marf proteins were detected. Expression levels of Opa1 and Marf proteins were elevated in flies fed a high-sucrose diet compared to those in control flies (Figure 2B). Thus, this result suggests that high-sucrose diet-induced Opa1 and Marf expression may induce excessive fusion of mitochondria in *Drosophila* nephrocytes, and this fly model could contribute to the identification and characterization of novel factors modulating mitochondrial function and morphology in the pathogenesis of DN.

## 4. Conclusions

This study revealed that a chronic high-sucrose diet induces morphological abnormalities in nephrocytes, as well as altered expression of Marf and Opa1, leading to disrupted mitochondrial dynamics in nephrocytes in an in vivo *Drosophila* model. This study further explores a new in vivo platform on which the imbalanced mitochondrial dynamics associated with a chronic high-sucrose diet can be investigated and novel regulators and therapeutic targets for modulation of mitochondrial dynamics in DN pathogenesis can be identified. The functional and structural similarities between *Drosophila* nephrocyte and human podocyte indicated that it could be useful as an in vivo model for studying the pathogenic mechanisms of diabetic nephropathy. Therefore, fly DN model can be probed genetically utilizing the *Drosophila* powerful genetic tools, and it can also offer an in vivo platform for drug screening that is amenable to the application of much larger synthetic or natural compound libraries. Furthermore, the use of high-throughput screens and studies of pharmacologic interventions for modulation of mitochondrial morphology in a *Drosophila* DN model have the potential to elucidate chemical mechanisms of drug action and to identify viable therapeutic interventions for human DN patients.

This study has some limitations. Because of the study aim of establishing a new *Drosophila* DN model for studying impaired mitochondrial dynamics in nephrocyte, our study did not include more detailed assessments of nephrocyte physiological function, such as the ultrastructure of nephrocyte slit diaphragms, the activity of hemolymph filtration, and information regarding urinary protein excretion. These in-depth assessments would be needed to draw more substantial conclusions on the pathological effects of enlarged mitochondria on nephrocyte function in this *Drosophila* DN model but are not available from the present study. Therefore, further studies are warranted to better clarify the exact molecular mechanisms of imbalanced mitochondrial dynamics in chronic kidney diseases.

## Data Availability

The data presented in this study are available on request from the corresponding author.
